# Conversion to Sirolimus Ameliorates Cyclosporine-Induced Nephropathy in the Rat: Focus on Serum, Urine, Gene, and Protein Renal Expression Biomarkers

**DOI:** 10.1155/2014/576929

**Published:** 2014-05-19

**Authors:** José Sereno, Sara Nunes, Paulo Rodrigues-Santos, Helena Vala, Petronila Rocha-Pereira, João Fernandes, Alice Santos-Silva, Frederico Teixeira, Flávio Reis

**Affiliations:** ^1^Laboratory of Pharmacology & Experimental Therapeutics, IBILI, Faculty of Medicine, University of Coimbra, Sub-Unit 1 (Pólo III), 3000-548 Coimbra, Portugal; ^2^Institute for Nuclear Sciences Applied to Health (ICNAS), University of Coimbra, 3000-548 Coimbra, Portugal; ^3^Institute of Immunology, Faculty of Medicine, University of Coimbra, 3004-504 Coimbra, Portugal; ^4^Immunology and Oncology Laboratory, Center for Neurosciences and Cell Biology, 3004-504 Coimbra, Portugal; ^5^ESAV, Polytechnic Institute of Viseu, 3504-510 Viseu, Portugal; ^6^Educational, Technologies and Health Study Center, Polytechnic Institute of Viseu, 3504-510 Viseu, Portugal; ^7^Research Centre for Health Sciences, Beira Interior University, 6201-506 Covilhã, Portugal; ^8^Biochemistry Department, Pharmacy Faculty, Porto University, 4050-313 Porto, Portugal; ^9^Institute for Molecular and Cellular Biology, Porto University, 4150-180 Porto, Portugal

## Abstract

Protocols of conversion from cyclosporin A (CsA) to sirolimus (SRL) have been widely used in immunotherapy after transplantation to prevent CsA-induced nephropathy, but the molecular mechanisms underlying these protocols remain nuclear. This study aimed to identify the molecular pathways and putative biomarkers of CsA-to-SRL conversion in a rat model. Four animal groups (*n* = 6) were tested during 9 weeks: control, CsA, SRL, and conversion (CsA for 3 weeks followed by SRL for 6 weeks). Classical and emergent serum, urinary, and kidney tissue (gene and protein expression) markers were assessed. Renal lesions were analyzed in hematoxylin and eosin, periodic acid-Schiff, and Masson's trichrome stains. SRL-treated rats presented proteinuria and NGAL (serum and urinary) as the best markers of renal impairment. Short CsA treatment presented slight or even absent kidney lesions and TGF-**β**, NF-**κ** **β**, mTOR, PCNA, TP53, KIM-1, and CTGF as relevant gene and protein changes. Prolonged CsA exposure aggravated renal damage, without clear changes on the traditional markers, but with changes in serums TGF-**β** and IL-7, TBARs clearance, and kidney TGF-**β** and mTOR. Conversion to SRL prevented CsA-induced renal damage evolution (absent/mild grade lesions), while NGAL (serum versus urine) seems to be a feasible biomarker of CsA replacement to SRL.

## 1. Introduction

Calcineurin inhibitors, such as cyclosporin A (CsA), are clinically important immunosuppressive drugs for prevention of allograft rejection after organ transplantation and also for several autoimmune disorders, such as psoriasis, rheumatoid arthritis, systemic lupus erythematosus, and inflammatory bowel disease, among other indications [1, 2]. Despite the impressive reduction in the number of acute rejection episodes after the beginning of CsA use in clinical practice, long-term therapy is typically associated with drug-induced nephrotoxicity [3]. Renal dysfunction is an independent risk factor for graft loss and mortality after kidney transplantation (KTx) and cardiovascular disease (CVD) is the main cause of dead post-KTx [4–6]; thus, extended long-term graft survival has not been completely achieved.

Complete avoidance of CNIs, in particular of CsA, from transplantation immunotherapy, has been viewed as an invalid option by almost all the transplantation centres worldwide, particularly because of the risks in acute rejection. The main long-term goals of immunosuppressive therapy are to reduce drug exposure while maintaining a well-functioning graft, keeping efficacy and minimizing drug-induced serious side-effects, including infections and nephrotoxicity [7]. The recognition of these serious adverse effects sparked interest in CsA-sparing strategies [8]: dose reduction is associated with a modest improvement in renal function, but CsA-induced nephropathy is progressive over time when exposure is maintained; CsA avoidance is associated with high acute rejection rates and is not an option; minimization protocols are the current preferred therapy, including the conversion from CsA to other drugs, especially sirolimus (SRL), an inhibitor of the mammalian target of rapamycin (mTOR) [9–11]. Despite the SRL-evoked proteinuria, this drug has been indicated as a less nephrotoxic immunosuppressive agent per se when compared with calcineurin inhibitors [12], but its use after CsA would have an impact on the renal function/structure that should be precisely defined. The major question nowadays concerning the protocols of immunotherapy is to find the most adequate duration for CsA exposure and the proper moment for replacement by other less nephrotoxic drugs, such as SRL, in order to afford renoprotection without compromising the graft by a rejection episode.

Early diagnosis of nephropathy can greatly improve patient diagnosis, but the initial stages of CsA-induced nephropathy are largely asymptomatic, making early diagnosis difficult [13]. Since the current diagnostic techniques employed to detect CsA nephropathy seem to be unsatisfactory, the identification of novel, early disease indicators is currently a major research focus. Identifying drug safety liabilities or predictive biomarkers for drug-induced organ damage is of great value. Drug safety evaluation has mainly been based on biochemical and histopathological data, but transcriptional profiling has the promise of being able to detect toxicity objectively. In addition, gene expression changes associated with toxicity may also accurately and earlier assist our understanding on the mechanism of certain drug-induced toxicity [14, 15], which will be pivotal for drugs with a low therapeutic window, such as the immunosuppressive agents. The precise mechanisms and biomarkers, underlying transition from renal dysfunction to nephrotoxicity, deserve better elucidation; experimental studies have been important to improve the knowledge on this translational issue of clinical relevance.

The mechanisms underlying CsA-induced nephropathy have been debated for the last decades and are clearly viewed as having a multifactorial nature (including vasorelaxant/vasoconstrictor disequilibrium, oxidative stress, apoptosis, and proliferation/fibrosis) [16–20], as evolving and changing with the increased duration of exposure [3, 21, 22] and as modulated by influence on renal tissue gene expression [23–25]. With nephrotoxicity remaining a major contributing factor to late allograft damage, it is crucial to understand the impact on the kidney tissue of protocols of conversion from CsA to SRL and identify early biomarkers in order to improve the therapeutic strategies after transplantation, thus extending long-term graft survival by reducing cardiorenal mortality. Molecular studies on animal tissues are essential to elucidate these questions and emergent biomarkers of renal damage, such as NGAL, KIM-1, and CTGF, among others, would increase our knowledge of how to better manage this drug-related nephropathy.

We have previously shown, in a preliminary study using an experimental model for comparing CsA and SRL, that distinct mechanisms and players are involved in the effects of these drugs on the kidney tissue, in a moment when renal lesions are almost absent [26]. In addition, our previous data suggested that early conversion from CsA to SRL promotes a better cardiorenal profile than late conversion [27], whose mechanisms and biomarkers deserve now more elucidation. The current study intended to clarify the pathways of nephropathy evolution in a protocol of conversion from CsA to SRL in the rat, focusing on serum, urine, and renal (gene and protein) tissue samples, as well as to elucidate the involvement of several emergent biomarkers of renal damage which are putative candidates to act as players in the evolution from renal dysfunction to nephrotoxicity.

## 2. Materials and Methods

### 2.1. Animals and Treatments

Male Wistar rats (aged 11 weeks) were purchased from Charles River Laboratories (Barcelona, Spain) and housed (two animals per cage) in IVC racks, subjected to 12 h dark/light cycles and given standard laboratory rat chow (IPM-R20, Letica, Barcelona, Spain) and free access to tap water. Forty-two animals were divided into two groups, according to the period of the treatments. Eighteen animals were used in a protocol of 3-week treatments and divided in 3 groups: control (vehicle), cyclosporin A (5 mg/Kg/day of Sandimmun Neoral, Novartis Farma Produtos Farmacêuticos SA, Sintra, Portugal), and sirolimus (1 mg/kg BW/day of Rapamune, Laboratórios Pfizer Lda., Lisbon, Portugal). Twenty-four animals were used in a protocol of 9-week treatments and divided into 4 groups: control (vehicle), cyclosporin A, sirolimus, and conversion (cyclosporin A during 3 weeks and sirolimus during the last 6 weeks). Treatments were performed by oral gavage and body weight was monitored daily. Animal experiments were conducted according to the European Council Directives on Animal Care and to the National Authorities.

### 2.2. Sample Collection and Preparation

The rats were anesthetized (i.p.) with 2 mg/Kg BW of a 2 : 1 (v : v) 50 mg/mL Ketamine (Ketalar, Parke-Davis, Pfizer Laboratories Ltd, Seixal, Portugal) solution in 2.5% chlorpromazine (Largatil, Rhône-Poulenc Rorer, Vitória laboratories, Amadora, Portugal). When the animal did not present response to stimulus, blood samples were immediately collected by venipuncture from the jugular vein in needles with no anticoagulant for serum samples collection. Then, the rats were sacrificed by cervical dislocation, and the kidneys were immediately removed, weighted, divided, and stored according to the next procedure: RNA-stabilizer reagent for gene expression determinations, frozen in nitrogen for lipid peroxidation assays, prefixed with formaldehyde for histopathological analysis and immunohistochemical detections.

### 2.3. Serum and Urinary Measures

#### 2.3.1. Serum Biochemical Data

Serum creatinine and blood urea nitrogen (BUN) contents were evaluated by automatic validated methods and equipment (Hitachi 717 analyser, Roche Diagnostics Inc., MA, USA). Serum levels of interleukin 1*β* (IL-1*β*), interleukin 2 (IL-2), vascular epidermal growth factor (VEGF), and transforming growth factor beta 1 (TGF-*β*_1_) were measured by ultrasensitive Quantikine ELISA kits (R&D Systems, Minneapolis, USA). High sensitivity CRP (hsCRP) was detected by using an ELISA kit (Alpha Diagnostic International, San Antonio, USA). Interleukin-7 (IL-7) was measured through an ELISA kit obtained from Wuhan EIAAB Science Co (Wuhan, China).

#### 2.3.2. Urinary Data

The animals were housed in metabolic cages during 24 hours and received tap water and food* ad libitum*. The urine concentration of creatinine, BUN, and protein was assessed in 24-hour urine (Cobas Integra 400 plus, Roche), and the urine volumes were measured in order to calculate creatinine and BUN clearance and glomerular filtration rate, as previously described [28].

#### 2.3.3. Serum, Kidney, and 24-Hour Urine Lipid Peroxidation

Lipid peroxidation was determined by assaying the malondialdehyde (MDA) production by means of the thiobarbituric acid (TBA) test. Briefly, 100 *μ*L of kidney tissue supernatant, serum, or urine (previously centrifuged to remove particulates) was incubated 1 hour in a TBA solution. Samples incubated at 90°C for 60 min. In this test, one molecule of MDA reacts with two molecules of TBA with the production of a pink pigment producing maximal absorbance at 532 nm. The concentration of MDA was calculated with respect to a calibration curve using 1,1,3,3-tetramethoxypropane as the external standard (range: 0.1–83.5 *μ*M) and results were expressed as *μ*M/g of kidney tissue and *μ*M of serum or urine.

### 2.4. RT-qPCR Kidney Gene Expression

#### 2.4.1. Total RNA Isolation

The kidneys were stored in RNA later solution (Ambion, Austin, TX, USA). For RNA extraction, 10 mg of tissue was weighted, 450 *μ*L of RLT lysis buffer was added, and tissue disruption and homogenization for 2 min at 30 Hz were performed using a TissueLyser (Qiagen, Hilden, Germany). Tissue lysates were processed according to the RNeasy Mini Kit protocol (Qiagen, Hilden, Germany). Total RNA was eluted in 50 *μ*L of RNase-free water (without optional treatment with DNAse). In order to quantify the amount of total RNA extracted and to verify RNA integrity (RIN, RNA Integrity Number), samples were analyzed using a 6000 Nano Chip kit, in the Agilent 2100 Bioanalyzer (Agilent Technologies, Walbronn, Germany) and the 2100 expert software, following manufacturer's instructions. The isolation yield was from 0.5 to 3 *μ*g; RIN values were 6.0–9.0 and purity (A260/A280) was 1.8–2.0.

#### 2.4.2. Reverse Transcription

RNA was reverse transcribed with SuperScript III First-Strand Synthesis System for RT-PCR (Invitrogen, California, USA). One microgram of total RNA was mixed with a 2x First-Strand Reaction Mix and a SuperScript III Enzyme Mix (Oligo (dT) plus random hexamers). Reactions were carried out in a thermocycler Gene Amp PCR System 9600 (Perkin Elmer, Norwalk, CT, USA), 10 min at 25°C, 30 min at 50°C, and 5 min at 85°C. Reaction products were then digested with 1 *μ*L (2 U) RNase H for 20 min at 37°C and, finally, cDNA was eluted to a final volume of 50 *μ*L and stored at −20°C.

#### 2.4.3. Relative Gene Expression Quantification

Gene expression was performed using a 7900 HT Sequence Detection System (Applied Biosystems, Foster City, USA). A normalization step preceded the gene expression quantification, using geNorm Housekeeping Gene Selection kit for* Rattus norvegicus* (Primer Design, Southampton, UK) and geNorm software (Ghent University Hospital, Center for Medical Genetics, Ghent, Belgium) to select optimal housekeeping genes for this study [29]. Real-time PCR reactions used specific QuantiTect Primer Assays (Qiagen, Hilden, Germany) with optimized primers for transforming growth factor beta 1 (QT00187796), proliferating cell nuclear antigen (QT00178647), mechanistic target of rapamycin (QT00180586), nuclear factor kappa B (QT01577975), monoclonal antibody Ki-67 (QT00450786), and tumor protein p53 (QT00193522) as proliferative markers; vascular endothelial growth factor beta (QT01290163) as angiogenic marker; interleukin 1 beta (QT00181657), interleukin 2 (QT00185360), tumor necrosis factor (QT00178717), cyclooxygenase 2 (QT00192934), and C-reactive protein (QT00391650) as inflammatory markers. Endogenous controls were used for kidney [glyceraldehyde-3-phosphate dehydrogenase (QT00199633), actin beta (QT00193473), and topoisomerase I (QT01820861)]. A QuantiTect SYBR Green PCR Kit (Qiagen, Hilden, Germany) was used according to manufacturer's instructions. RT-qPCR reactions were carried out with 100 ng cDNA sample, primers (50–200 nM), and 1x QuantiTect SYBR Green PCR Master Mix. Nontemplate control reactions were performed for each gene, in order to assure nonunspecific amplification. Reactions were performed with the following thermal profile: 10 min at 95°C plus 40 cycles of 15 seconds at 95°C and 1 min at 60°C. Real-time PCR results were analyzed with SDS 2.1 software (Applied Biosystems, Foster City, USA) and quantification used the 2^−ΔΔCt^ method [30]. The results were obtained in CNRQ (calibrated normalized relative quantities).

### 2.5. Histopathological Analysis

#### 2.5.1. Haematoxylin and Eosin Staining

Samples were fixed in Bock's fixative and embedded in paraffin wax, and 4 *μ*m thick sections were mounted on glass slides and stained for routine histopathological diagnosis with haematoxylin and eosin (H&E).

#### 2.5.2. Periodic Acid of Schiff Staining

Periodic acid of Schiff (PAS) was used to evaluate and quantify the renal lesions. Samples were fixed in 10% neutral formalin, embedded in paraffin wax, and 4 *μ*m thick sections were immersed in water and subsequently treated with a 1% aqueous solution of periodic acid, then washed to remove any traces of the periodic acid, and finally treated with Schiff's reagent. All samples were examined by light microscopy using a Zeiss Microscope Mod. Axioplan 2. The degree of injury visible by light microscopy was scored in a double-blinded fashion by two independent pathologists. Lesions were evaluated on the total tissue on the slide.

#### 2.5.3. Analysis of Lesions

Glomerular damage was assessed by evaluating mesangial expansion, the glomerular basement membrane and the Bowman's capsule thickenings, nodular sclerosis, and vascular pole hyalinosis. The analysed tubulointerstitial lesions comprised inflammatory infiltration, presence of hyaline cylinders, tubular basement membrane irregularity, tubular calcification, tubular vacuolization, and the association of interstitial fibrosis and tubular atrophy (IFTA). The evaluation of vascular lesions was concentrated on vascular congestion and hyperemia, arteriolar vacuolization, arteriolosclerosis, and arteriosclerosis. A semiquantitative rating for each slide ranging from normal (or minimal) to severe (extensive damage) was assigned to each component. Severity was graded as absent/normal (0), mild (1), moderate (2), and severe (3). Scoring was defined according to the extension of the lesion (number of capsules): normal: 0%; mild: <25%; moderate: 25–50%; severe: >50%. The final score of each sample was obtained by the average score observed in the individual glomeruli, in the considered microscopic fields. Tubular calcification was evaluated and graded by the same semiquantitative method. Regarding vascular lesions, arteriosclerosis was scored as 0 if no intimal thickening was present, as 1 if intimal thickening was less than the thickness of the media, and as 2 if intimal thickening was more than the thickness of the media and considering the worst artery on the slide. Using PAS, the rating was set for intensity and extension of staining, ranging from 0 (no staining) to 3 (intense and extensive staining), respecting tissue specificity scoring when adequate.

#### 2.5.4. Masson's Trichrome Staining

Deparaffinise and rehydrate through 100% alcohol, 95% alcohol, and 70% alcohol. Wash in distilled water. After that, refix in Bouin's solution for 1 hour at 56°C to improve staining quality and rinse in running tap water for 5–10 minutes to remove the yellow colour. Stain in Weigert's iron hematoxylin working solution for 10 minutes and rinse in running warm tap water for 10 minutes. Wash in distilled water. Stain in Biebrich scarlet-acid fuchsin solution for 10–15 minutes and wash in distilled water. Differentiate in phosphomolybdic-phosphotungstic acid solution for 10–15 minutes and transfer sections directly to aniline blue solution and stain for 5–10 minutes. Rinse briefly in distilled water and differentiate in 1% acetic acid solution for 2–5 minutes and wash in distilled water. Finally, dehydrate very quickly through 95% ethyl alcohol, absolute ethyl alcohol, clear in xylene, and mount with resinous mounting medium. All samples were examined in a blind fashion by expert personnel (pathologists) by light microscopy using a Zeiss Microscope Mod. Axioplan 2.

### 2.6. Immunohistochemical Analysis

Immunohistochemical analyses were performed in 4 *μ*m thick sections with sagittal orientation of kidney fixed in Bock's fixative and embedded in paraffin wax. The samples were processed by indirect immune detection technique with mouse and rabbit specific HRP/DAB detection IHC kit (Abcam, Cambridge, UK) using the primary antibody mammalian target of rapamycin (Millipore Corporation, Billerica, MA, USA, 04-385) (dilution 1 : 250). The protocol was executed according to the manufacturer's instructions. In this study, we employed primary antibodies against CTGF (dilution 1 : 100, ab6992; Abcam), TGF-b (dilution 1 : 100, ab66043; Abcam), mTOR (dilution 1 : 250, 04-385; Millipore), NF-*κβ* p50 (dilution 1 : 500, sc-114; Santa Cruz Biotechnology), and KIM-1 (dilution 1 : 14, AF3689; R&D Systems). For KIM-1 detection the secondary antibody was anti-goat (dilution 1 : 500, sc2771; Santa Cruz Biotechnology). After testing the different antigen-retrieval methods and negative controls, immunohistochemical procedures were optimized. To identify PCNA protein we used a standard kit (ready to use, 93-1143, Invitrogen Corporation). An appropriate positive control was used in each staining run, and each slide was stained with a negative control obtained by omitting the primary antibody. Standard procedures were used for visualisation and the staining was quantified using a semiquantitative scale (1–4) that evaluated both the intensity and area of staining. Intensity was graded as very low (1), low (2), moderate/mild (3), and high (4); staining area was graded as <25% (1), 25–50% (2), 25–75% (3), and >75% (4). All slides were reviewed independently by 2 investigators blinded to the data. In this evaluation a quantitative immunohistochemical score (QIC) was calculated. QIC = % of staining area * staining intensity * 0.1.

### 2.7. Statistical Analysis

Statistical analyses were performed using the GraphPad Prism for Windows (version 5.00). The results are presented as means ± S.E.M. Comparisons between groups were performed using one-way ANOVA test, followed by the post hoc Bonferroni's multiple comparisons. The association between categorical variables was analyzed using Pearson's test in the IBM Statistical Package for Social Sciences (SPSS) for Windows, version 20.0 (SPSS Inc., Chicago, IL, USA). Significance was accepted at *P* less than 0.05.

## 3. Results

### 3.1. Kidney Histomorphological Changes and Collagen Deposition

**Figure 1:**
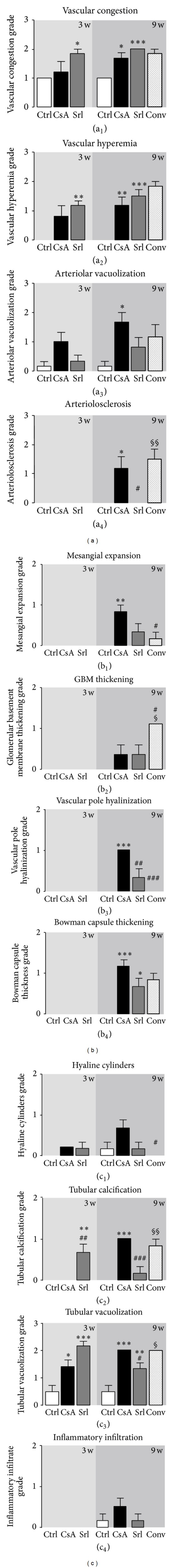
Semiquantitative evaluation of vascular (a), glomerular (b), and tubulointerstitial (c) lesions. Each graphic represents one lesion for the 3 groups at week 3 (control, cyclosporin A, and sirolimus) and 4 groups at week 9 (control, cyclosporin A, sirolimus, and conversion). Values are mean ± SEM. *P < 0.05, **P < 0.01, and ***P < 0.001 versus control; ^#^ *P* < 0.05 and ^###^ *P* < 0.001 versus cyclosporin;  ^§^ *P* < 0.05 and  ^§§^ *P* < 0.01 versus sirolimus. GBM thickening, glomerular basement membrane thickening.

**Figure 2:**
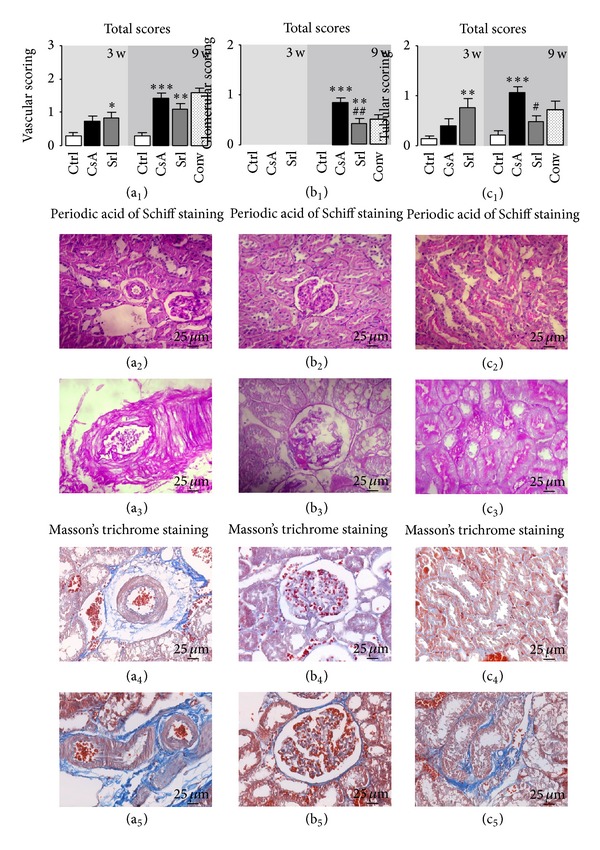
Total score of vascular (a_1_), glomerular (b_1_), and tubulointerstitial (c_1_) lesions and representative photomicrographs of kidney histomorphologic sections with PAS and Masson's trichrome stainings. The image (a_2_) represents a normal kidney arteriole from the control group and A_3_ an arteriolosclerosis lesion present in all the rats treated with CsA. (b_2_) and (b_3_) represent a normal capsule and a vascular pole hyalinization and Bowman's capsule thickening from the CsA group, respectively. (c_2_) and (c_3_) images match normal tubules and tubular calcification in the kidney of CsA-treated rats, respectively. Representative photomicrographs of kidney histomorphologic sections with Masson's trichrome staining for control ((a_4_), (b_4_), and (c_4_)) and CsA ((a_5_), (b_5_), and (c_5_)). CsA promotes collagen fibers deposition around arterioles, Bowman's capsules, and tubules (fibrosis). Values are mean ± SEM. *P < 0.05, **P < 0.01, and ***P < 0.001 versus the control group; ^#^ *P* < 0.05 and ^##^ *P* < 0.01 versus cyclosporin.

Nephrotoxicity was confirmed by two independent pathologists, which have characterized the lesions through the attribution of degrees to each vascular, glomerular, and tubular lesion, examining kidney slices stained with H&E and PAS. After 3 weeks of CsA treatment, only slight morphological changes on the tubules (tubular vacuolization) were found when compared with the control (Figure 1). However, signs of toxicity were identified in the vessels since some kidney slices revealed arteriolar vacuolization and hyperemia. SRL treatment during identical period, described in the literature as less nephrotoxic than CsA, surprisingly revealed some lesions in the vascular (congestion and hyperemia) and tubular (vacuolization and calcification) fields. Total lesion scoring showed that only SRL was able to induce significant damage in the vessels (*P* < 0.05) and tubules (*P* < 0.01) after the first 3-week period (Figures 2(a_1_) and 2(c_1_)). Long-term CsA treatment (9 weeks) promoted relevant changes on the kidney (vessels, glomeruli, and tubules) structure, which are viewed as clear signs of nephrotoxicity. The main changes encountered compared with the normal controls are represented in Figure 1. In the long-term CsA exposure, vascular congestion, vascular hyperemia, and arteriolar vacuolization and arteriolosclerosis were identified, being all statistically significant versus the control group (Figure 1(a_1–4_)). Sirolimus revealed similar pattern to that found for CsA but does not induce arteriolosclerosis, compared to CsA (*P* < 0.05). The conversion protocol does not promote any advantage in the vascular field when compared with the CsA treatment alone; two rats of the group presented arteriosclerosis (grades 1 and 2).

Regarding the glomerular field after 9 weeks of CsA treatment, the major lesions found were mesangial expansion (*P* < 0.01), hyalinosis of vascular pole (*P* < 0.001), and thickening of Bowman's capsule (*P* < 0.001) when compared with the control rat kidneys (Figure 1(b_1–4_)). One rat presented hydronephrosis and cortical atrophy. In the SRL treatment the single significant lesion found was Bowman's capsule thickening, confirming a better profile in the conversion protocol. Mesangial expansion and vascular pole hyalinization grade were almost absent when compared with the isolate CsA treatment. However, all rats from the conversion group showed glomerular basement membrane thickening (*P* < 0.05 versus CsA). The global glomerular score clearly showed that SRL is less toxic than CsA (*P* < 0.01) and this was reflected in the lower score found in the conversion group.

CsA induces tubular damages and the main lesions identified were tubular vacuolization and calcification (*P* < 0.001, both), versus the normal profile found in the control rats (Figures 1(c_1–4_) and 2(c_2-3_)). However, the presence of hyaline cylinders and inflammatory infiltration was identified in almost all the kidneys (grade 1, less than 25% of the tubules). Sirolimus treatment only induced tubular vacuolization. However, when CsA was used prior to SRL (conversion group), tubular calcification and vacuolization remain present in the same grade than that encountered for CsA therapy alone. In contrast, hyaline cylinders (*P* < 0.05) and inflammatory infiltration were absent in the kidneys of the conversion group rats.  Figure 2(c_1_) gives an idea about the treatments influence on the tubules. Clearly, CsA promotes more tubular damage than SRL, and CsA conversion to SRL revealed less total lesions grade in glomerular and tubular fields.

Collagen is the major insoluble fibrous protein in the extracellular matrix and in connective tissue and is clearly marked with Masson's trichrome; modifications of collagen production reflect cellular changes and consequent kidney dysfunction. In the kidneys from vehicle-treated rats, collagen staining was rare in the glomeruli, and a small amount of blue Trichrome staining appeared in the outer borders tubules and around the vessels (Figures 2(a_4-5_), 2(b_4-5_), and 2(c_4-5_)). After 9 weeks of CsA treatment, staining was clearly visible in the outer borders of tubular cells (cortex and medulla), well representing wide-spread interstitial fibrosis. Bowman's capsule thickening also occurred in some glomeruli; around the vessels we also verified higher collagen deposition in the CsA-treated rats. Sirolimus and conversion group revealed normal collagen staining (comparable to that encountered in the control group).

### 3.2. Nephrotoxicity Evaluation through Serum, Kidney, and Urine Markers

**Figure 3:**
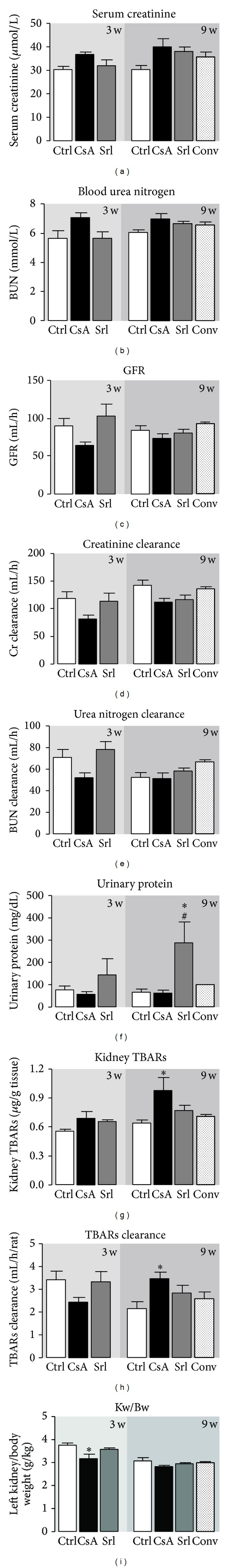
Serum, urine, and kidney markers of renal function. Creatinine serum levels (a) and clearance (d), blood urea nitrogen levels (c) and clearance (f), glomerular filtration rate (c) and urinary protein (f), kidney lipid peroxidation (malondialdehyde levels) (g), malondialdehyde clearance (h), and kidney weight/body weight ratio (i), at week 3 and week 9 for all treatments. Values are mean ± SEM. *P < 0.05 versus control; ^#^ *P* < 0.05 versus cyclosporin.

Classical serum markers of renal function, such as creatinine and BUN, presented a trend to increased levels after 3 weeks of CsA treatment, accompanied by a trend to decreased creatinine and BUN clearances (Figures 3(a), 3(b), 3(d), and 3(e)), resp.); however, all those measures did not reach a statistical significant value. Moreover, CsA showed a trend to decreased glomerular filtration rate (GFR). On the other hand, unchanged values were found for the SRL group for all serum and urine markers. Long-term CsA treatment (9 weeks) presented a trend to aggravated serum creatinine and BUN levels; in addition, while GFR and kw/bw remained decreased, kidney TBARs production and clearance significantly increased (*P* < 0.05). The main change found for the SRL treatment after 3 weeks was increased urinary protein, with additional increment after 9 weeks, suggesting a time-dependent effect. The conversion protocol revealed no significant change on serum creatinine and BUN levels and clearances; moreover, GFR remains unchanged as well as urinary protein and TBARs (Figure 3).

**Figure 4:**
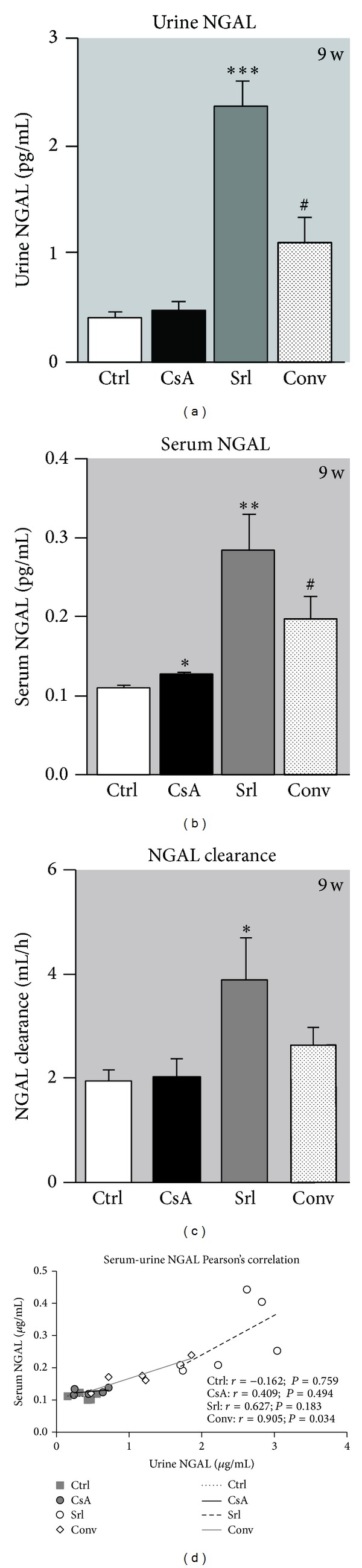
Serum and urine NGAL correlation as kidney marker of toxicity. Urine (a), serum NGAL (b), and clearance (c) at week 9. Values are mean ± SEM. Correlation and *P* value of urine and serum NGAL are shown in (d). *P < 0.05, **P < 0.01, and ***P < 0.001 versus the control group; ^#^ *P* < 0.05 versus cyclosporin A.

NGAL has been described as a putative biomarker of nephrotoxicity. Long-term CsA treatment only promoted serum NGAL increment (*P* < 0.05), without affecting urine and clearance values. The rats treated with SRL presented increased serum and urine NGAL contents, as well as augmented clearance. The use of SRL to replace CsA leads to increased NGAL levels in serum and urine compared to the CsA group (*P* < 0.05) (Figures 4(a), 4(b), and 4(c)).  Figure 4(d) showed the correlation between serum and urine NGAL levels; interestingly, with only 6 samples of each group, while the control and CsA groups were unable to show significant Person's correlation; in SRL group there was a good linearity (*r* = 0,627, *P* = 0,183), which was even more evident and statistically significant, in the conversion group (*r* = 0.905, *P* = 0.034).

### 3.3. Serum Markers of Inflammation, Proliferation, and Angiogenesis

**Figure 5:**
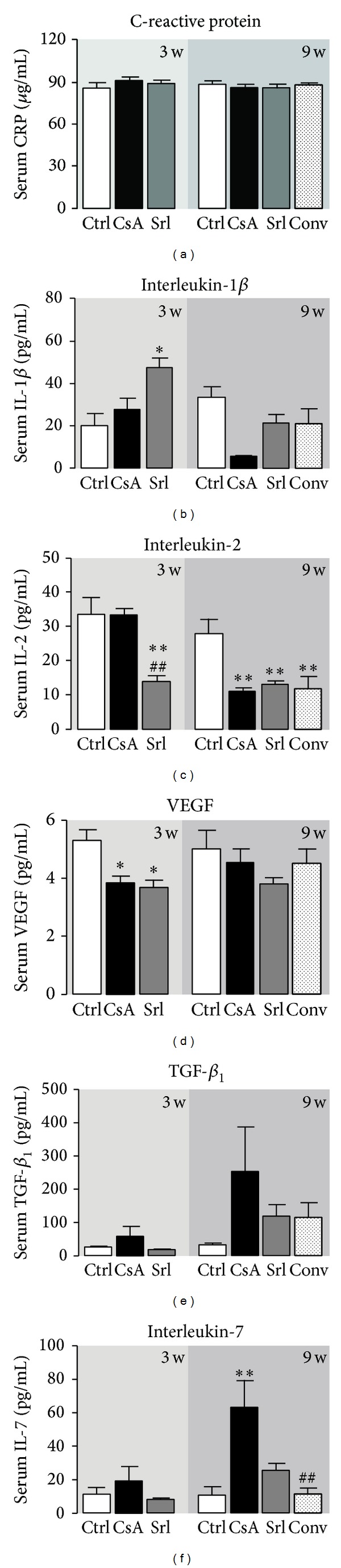
Serum markers of inflammation, proliferation, and angiogenesis. C-reactive protein (a), interleukin-1*β* (b), interleukin-2 (c), vascular endothelial growth factor (d), transforming growth factor beta-1 (e), and interleukin-7 (f). Values are mean ± SEM. *P < 0.05 versus control; ^#^ *P* < 0.05 versus cyclosporin A.

Unchanged values of serum hsCRP were found for both immunosuppressive drugs when compared with the control group. However, serum IL-1*β* showed distinct patterns; in fact, SRL treatment was able to increase IL-1*β* levels in the short-term treatment and CsA to decrease in the long-term use (Figures 5(a) and 5(b)). Serum IL-2 levels, which is simultaneously a marker of inflammation and immunosuppressive activity, decreased after 3 weeks of SRL treatment (*P* < 0.01). Identical reduction (*P* < 0.01) was found at 9 weeks for all the treated groups (CsA, SRL, and conversion) versus the control one (Figure 5(c)). Serum contents of the VEGF only decreased in the short-term treatment for both drugs, with unchanged values in long-term protocols. Serum TGF-beta levels showed a trend to increased values after 3 weeks, which was even more pronounced after 9 weeks in the CsA group (Figures 5(e) and 5(f)). Interestingly, similar pattern was encountered for serum IL-7 levels, showing significant correlation with serum TGF-*β*_1_ contents in the short- and long-term treatments (*r* = 0.871, *P* = 0.129; *r* = 0.873, *P* = 0.053, resp., in Person's Test).

### 3.4. Kidney Gene Expression Evaluation

**Figure 6:**
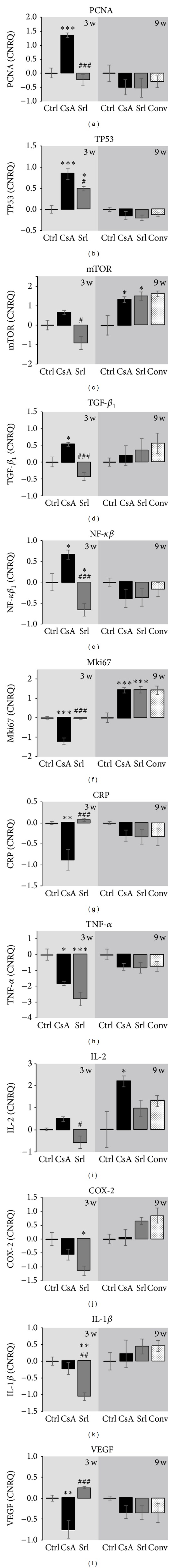
Kidney mRNA expression of proliferation, inflammation, and angiogenesis mediators. PCNA (a), TP53 (b), mTOR (c), TGF-*β*_1_(d), NF-*κ*B (e), and Mki67 (f) as proliferation status markers; CRP (g), TNF-*α* (h), IL-2 (i), COX-2 (j), and IL-1*β* (k) as inflammation status markers and VEGF (l) as angiogenesis status marker. Values are mean of CNRQ (calibrated normalized relative quantities) of the control ± SEM. *P < 0.05, **P < 0.01, and ***P < 0.001 versus control; ^#^ *P* < 0.05, ^##^ *P* < 0.01, and ^###^ *P* < 0.001 versus cyclosporin A. COX-2, ciclooxigenase-2; CRP, C-reactive protein; IL-1*β*, interleukin-1 beta; IL-2, interleukin-2; MKi67, antigen identified by monoclonal antibody Ki-67; mTOR, mammalian target of rapamycin; NF-*κ*B, nuclear factor kappa B; PCNA, proliferating cell nuclear antigen; TGF-*β*_1_, transforming growth factor beta 1; TNF-*α*, tumor necrosis factor alpha; TP53, tumor protein p53; VEGF, vascular epidermal growth factor.

Several markers of proliferation, fibrosis, inflammation, and angiogenesis were evaluated in terms of kidney mRNA expression in the weeks 3 and 9 for the three immunosuppressive protocols in comparison to control group (Figure 6). After 3 weeks of CsA treatment, a significant downregulation of the antigen identified by the monoclonal antibody Ki67 (MKi67) (*P* < 0.001), CRP (*P* < 0.01), TNF-alpha (*P* < 0.05), and VEGF (*P* < 0.01) was found (Figure 6). However, IL-2, COX-2, mTOR, and IL-1*β* remain unchanged. Furthermore, there was a significant overexpression of proliferating cell nuclear antigen (PCNA) and tumor protein p53 (TP53) mRNA (*P* < 0.001), accompanied by a slight increase (*P* < 0.05) in the expression of TGF-*β*_1_ and NF-*κ*B. On the other hand, the mTOR inhibitor only stimulated the expression of TP53 gene and downregulated some inflammatory markers (TNF-*α*, COX-2, and IL-1*β*). In the long-term CsA treatment, almost all the genes presented normal mRNA expression, when compared with the control group. However, a significant upregulation of IL-2, mTOR, and Mki67 was encountered. In the SRL and conversion groups only mTOR and Mki67 remained overexpressed, in contrast to what was observed with the short-term exposures.

### 3.5. Kidney Protein Expression Evaluation

**Figure 7:**
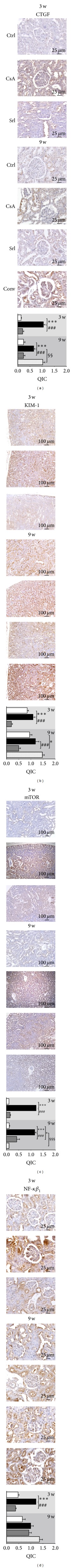
Kidney protein expression by immunohistochemistry. CTGF (a), KIM-1 (b), mTOR (c), and NF-*κ*B_1_ (d). Each figure is representative of the groups at week 3 (control, cyclosporin A, and sirolimus) and 4 groups at week 9 (control, cyclosporin A, sirolimus, and conversion). CTGF, connective tissue growth factor; KIM-1, kidney injury molecule-1; mTOR, mammalian target of rapamycin; NF-*κ*B_1_, nuclear factor kappa beta-1.

**Figure 8:**
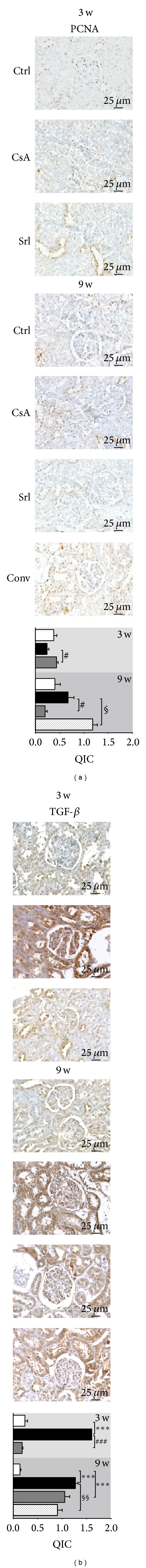
Kidney protein expression by immunohistochemistry. PCNA (a) and TGF-*β*_1_ (b). Each figure is representative of the groups at week 3 (control, cyclosporin A, and sirolimus) and 4 groups at week 9 (control, cyclosporin A, sirolimus, and conversion). PCNA, proliferating cell nuclear antigen; TGF-*β*_1_, transforming growth factor beta 1.

Short-term CsA treatment increased CTGF, KIM-1, mTOR, NF-*κβ*_1_, and TGF-*β* (*P* < 0.001) protein expression, when compared to control, while SRL treatment was unable to promote changes on the expression of these proteins (Figures 7 and 8). Long-term treatment with CsA promoted increased expression of mTOR, TGF-*β*, and CTGF, versus the control group, but the last two proteins present less area and stain intensity (QIC score) when compared to the short-term (3 weeks) CsA treatment. Kidney KIM-1, NF-*κβ*_1_, and PCNA expression were unchanged in the CsA-treated rats when compared with the control animals. SRL treatment promoted, after 9 weeks, a decreased KIM-1 expression and overexpression of TGF-*β*. In the conversion protocol, CTGF and PCNA protein overexpression was obtained, but mTOR and TGF-*β* expression were significantly reduced when compared with the CsA group (Figures 7 and 8).

## 4. Discussion

Monitoring immunosuppressive therapy in solid organ transplant patients is based on measuring putative indicators of allograft rejection, as well as on regularly assessing drug blood levels, which should be maintained within the established therapeutic range for the drug in order to maintain immunosuppressive efficacy without excessive/undesirable side-effects. Drug-related nephrotoxicity evaluation has been mainly based on classical serum measures of renal function, which are easier to perform and less expensive; however, an increasing amount of evidence suggests that these markers cannot accurately reflect the renal function status at a given time point of drug use. In fact, traditional markers of nephrotoxicity, such as increased BUN or serum creatinine, have been reported as insensitive, only indicating damage when 70–80% of renal epithelial mass has been lost [31, 32]. The use of noninvasive samples (e.g., urine) has been pointed as a choice to access drug-related toxicity; however, detection of enzymes and other proteins can be difficult due to their instability and high variability levels in urine [32]. Identifying potentially useful biomarkers in peripheral blood and urine, compared to kidney tissue markers (gene or protein), will be clinically very important. The current study was intended to clarify the pathways of nephropathy evolution in a protocol of conversion from CsA to SRL in the rat, focusing on serum, urine, and renal (gene and protein) tissue samples, as well as to elucidate the involvement of several emergent biomarkers of renal damage which are putative candidates to act as players in the evolution from renal dysfunction to nephrotoxicity.

In our study, the classical serum and urine markers were unable to accurately reflect the changes on renal function after both the short- and long-term treatments, despite the presence of renal lesion, which were more pronounced for the longer CsA exposure; that failure demonstrates the need of better biomarkers of renal dysfunction/damage. Regarding renal pathology characterization, we found that vessels are the first renal structures affected by CsA use, after just 3 weeks of treatment, as shown by the presence of some lesions, such as vascular hyperemia and arteriolar vacuolization (that might be related to hypertension appearance); the lesions were further aggravated with prolonged CsA exposure. This data complements the information that the first CsA pathologic events are related to afferent arteriolar vasoconstriction, thrombotic microangiopathy, and isomeric tubular vacuolization [33]. In addition, acute CsA events are related to decreased vasodilation and unopposed vasoconstriction and free radical formation, which are among the main mechanisms underlying development of hypertension and decreased GFR [34]. SRL has been described as a less nephrotoxic agent than CsA [20], which explains the fact that mTOR inhibitors have been used to replace CsA [9, 11]. According to our data, SRL induces less toxicity in vascular, glomerular, and tubular fields than CsA, and this factor leads to a better profile in the conversion group; however, tubular vascular congestion and hyperemia were not prevented when using SRL after CsA. Overall, we can conclude that total vascular scoring in the conversion group remained similar to the CsA group, but glomerular and tubular lesions scores were clearly reduced due to conversion to SRL. In addition, in the CsA-treated rats there was development of kidney fibrosis through collagen formation and deposition around vessels and tubules, together with bowman's capsules thickening. SRL, per se, or even after CsA treatment, was unable to present fibrosis or collagen deposition.

After 3 weeks of CsA exposure very slight changes on tissue structure were found, with absent or only mild lesions; however, after the long-term CsA exposure, significant glomerular, tubular, and vascular lesions were observed. In spite of that, at week 9 renal markers used in clinical practice (GFR, creatinine and BUN contents and clearances) appeared only modestly changed. Additionally, we observed an interesting variation of MDA clearance levels between 3 and 9 weeks of CsA treatment, when renal lesions were clearly noted. Knight et al. detected high MDA levels in urine of transplanted patients, but they were unable to explain their importance [35]. Our data suggests that MDA clearance could be a predictive marker of CsA-induced nephrotoxicity, as increased MDA clearance appears at the same time point as the first kidney lesions. Oxidative stress can promote the formation/release of a variety of vasoactive mediators [36] that can affect renal function directly by causing renal vasoconstriction or decreasing the glomerular capillary ultrafiltration coefficient, thus reducing the GFR. Moreover, the relationship between proteinuria and CsA-evoked nephrotoxicity is complex, limiting its power as an early marker [13]. Lipid peroxidation occurs as a result of multiunsaturated lipids reacting with oxidizing agents, promoting oxidative stress in the kidney structures. Urinary MDA reflects the presence of renal damage, which may be the cause or the consequence of lipid peroxidation, and the correlation between MDA clearance and kidney lesion grade could be a good strategy to identify early CsA-induced nephrotoxicity. The presence of slight or low grade lesions on the chronic SRL treatment and in the conversion protocol groups reinforces this idea, because no significance increase was found in MDA clearance for both groups.

The development of noninvasive biomarker that could diagnose renal dysfunction early and also monitor the response to therapy, as well as the ability to predict severity and outcome, would be very valuable. It is also important to recognize that changes in serum creatinine and BUN concentrations primarily reflect functional changes in filtration capacity and are not genuine injury markers [37]. During the last years, there has been an effort to identify better accurate biomarkers of acute CsA-induced nephrotoxicity. Gelatinase-associated lipocalin (NGAL) has been indicated as an acute marker of nephrotoxicity [38, 39]. NGAL in urine and plasma could have a 10,000-fold and 100-fold concentration rise, respectively, from normal levels in cases of renal injury. This could make NGAL a potentially very sensitive marker of different degrees of renal wound. However, according to our data, short- and long-term CsA treatments (clearly described as a nephrotoxic drug) were unable to promote increased serum and urine NGAL levels. Curiously, urine and serum samples presented linearity in the SRL group and a strong correlation in the conversion group. SRL is described in the literature as a less nephrotoxic agent than the calcineurin inhibitors, but one of the effects better described is the development of SRL-evoked proteinuria [40, 41]. The elevation in urine and serum NGAL levels in the SRL-treated rats in our study could be related to the proteinuria appearance. Recently, a mouse mTOR knockout model revealed accumulation of autolysosomal vesicle in podocytes that potentiated proteinuria appearance [41] and reduced AKT activity, thus affecting podocyte cytoskeleton [42]. Moreover, concerning the tubular field, mTOR inhibition by using rapamycin has a role in the protein transport because it reduces tubular protein reabsorption that contributes to increasing urinary protein levels [43]. Furthermore, angiotensin II receptor blocker can counteract the effect of sirolimus, not only through hemodynamic changes but also partly by repairing the injury of podocytes [40].

In our study, a trend to increased serum and kidney TGF-*β*_1_ was found in the CsA-treated rats, starting after just 3 weeks and aggravating with prolonged exposure, suggesting this factor as a putative good biomarker of nephrotoxicity progression. Interestingly, similar pattern was encountered for serum IL-7 levels, showing significant correlation with serum TGF-*β*_1_ contents in the short- and long-term treatments. IL-7 is produced constitutively by stromal cells and consumed by the available pool of resting T cells, all of which express the IL-7 receptor (IL-7R) at high levels except for CD4^+^ CD25^+^ regulatory T cells. Circulating IL-7 levels increase during periods of lymphopenia to maintain naïve T-cell homeostasis and support the thymic-independent peripheral expansion and maintenance of mature T cells [44] because they upregulate bcl-2 protein that has antiapoptotic properties [45, 46]. CsA treatment decreases the immune system, specially T-cells number and activation, but alternatively the remaining immune cells can counterwork the immunologic depression by increasing IL-7 levels which could be correlated with the progression of chronic kidney disease in this study.

Changes in messenger RNA expression are considered to be one of the earliest events, which may occur in response to cellular and tissue damage; it has been speculated that these biomarkers might help to predict adverse effects before damage is indicated by the current gold standard markers (clinical chemistry and histopathology). Current theories point that renal damage is caused by nonimmunological factors, such as ischemia, which lead to activation of various proinflammatory and profibrotic mediators. A parallel concept of how CsA might induce renal injury was described by Li and Yang, suggesting that kidney damage involves activation of the innate immune response that causes NF-*κβ* activation and induces dendritic cell maturation and T-lymphocyte infiltration into the graft, with both pathways ultimately resulting in interstitial inflammation and interstitial fibrosis that contributes to chronic nephropathy [47]. In agreement, our data confirm that CsA toxicity might start with increased NF-*κβ* gene (RT-qPCR) and protein (immunohistochemistry) overexpression after 3 weeks of CsA treatment, an effect that is then downregulated with prolonged exposure. SRL treatment reduced mRNA levels, resulting in normal protein expression, when compared to control, both in short- and long-term treatments, indicating that this nuclear factor could have an important impact in the development of nephrotoxicity. At the end of the conversion protocol (CsA replaced by SRL), no difference was found in gene expression but protein overexpression remained in the tubulointerstitial region, most probably because of the previous CsA exposure. In our animal model, the short-term CsA treatment was mainly associated with upregulation of TGF-*β*_1_ and PCNA in the kidney tissue, which has been identified as the key mediator of fibrosis and proliferation [47, 48]. However, these changes were accompanied by a putative compensatory response, since markers of inflammation (including COX2, TNF-*α*, and CRP), as well as of cellular proliferation (MKi67) and angiogenesis (VEGF), were downregulated, perhaps responsible for the attenuation of the cytotoxic effects of CsA in the short term. The overexpression of NF-*κβ* and TP53 might be included in this compensatory response, since they inhibit mTOR [49]. Short-term SRL treatment revealed acute anti-inflammatory, antifibrotic, and antiproliferative properties, viewed by the downregulation of kidney mRNA levels of TNF-*α*, COX2, IL-1*β*, TGF-*β*_1_, NF-*κβ*, and mTOR. Nevertheless, during prolonged CsA exposure, nephrotoxicity evolves, as viewed by the degree of increased histological lesions, which seems to be associated with other molecular pathways and mediators. In fact, there was a significant overexpression of MKi67, contrary to what was observed after the short-term treatment, suggesting a depletion of counter-regulatory responses, which was accompanied by a parallel increase in mTOR expression, a serine/threonine protein kinase, important in regulating cell growth, proliferation, motility, survival, protein synthesis, and transcription [48]. As Lieberthal and Levine demonstrated, mTOR plays an important role in mediating the process of regeneration and recovery, depending on the kidney damage extension [50]. Moreover, mTOR activity is low or absent in the normal kidney but increases markedly after acute kidney injury. In agreement, mTOR inhibition has been associated with amelioration of kidney fibrosis, glomerulosclerosis, and interstitial inflammation, having an important role in distinct renal diseases [50–52]. In our study, protein expression assessed by immunostaining revealed increased mTOR in the CsA-treated rats, which is in agreement with a previous study that suggested mTOR overexpression in CsA-treated rats, resulting in podocyte epithelial to mesenchymal transition leading to glomerular damage [53]. In addition, while normal kidney mTOR expression was found in the SRL-treated rats, there was an important decreased kidney expression in the conversion protocol group, which might explain the reduced lesions found when compared with the CsA monotherapy group.

In the last years, some toxicological studies showed hypothetical biomarkers that could predict acute nephropathy [32, 37, 54]. However, those studies were unable to assess if they could be viewed also as markers of chronic toxicity. Due to its functional reserve, minor effects on kidney function are too difficult to detect. Kidney injury molecule-1 (KIM-1) is a type 1 transmembrane protein expressed in the proximal tubules and further excreted in the urine; in the last years, KIM-1 has been pointed as a possible marker of renal injury in acute models. This factor has a role in proliferation and tissue repair [32, 55] because it confers phagocytic capacity to clear cell debris [56]. In our study, KIM-1 staining occurred in proximal tubule epithelial cells and might putatively be indicated as one of the most sensitive markers of tissue injury, in agreement with the previous suggestion of Rached et al. when studying nephrotoxin ochratoxin A [32]. In our study, after 3 weeks of CsA treatment, intense KIM-1 staining was found in the proximal tubules, but not after 9 weeks, when less stain intensity was found in all proximal tubules, suggesting that KIM-1 could be viewed as a putative good marker of acute CsA toxicity (without structural lesions), but not as a biomarker of chronic CsA treatment nephrotoxicity. KIM-1 is downexpressed in the kidneys of SRL-treated rats; however, when SRL was used to replace CsA (conversion group), a similar expression was found to that encountered for the CsA-treated rats after 3 weeks, suggesting that previous CsA exposure damaged some proximal tubules in an irreversible manner.

Connective tissue growth factor (CTGF) is a polypeptide implicated in the extracellular matrix synthesis that belongs to a profibrotic signalling (TGF-*β*_1_ downstream modulator) and has been pointed as a possible biomarker of CsA-evoked damage. In our model, kidney CTGF expression increased after short- and long-term treatment with CsA, in agreement with the kidney overexpression of TGF-*β*_1_ viewed by immunohistochemistry. After a longer CsA exposure the kidney expression of CTGF was slightly reduced, which might be explained by an increased urinary elimination, as previously suggested by O'Connell et al. in another experimental study [18]. SRL treatment per se does not promote any significant CTGF expression when compared with the control, in agreement with the absence of fibrosis or collagen deposition in the SRL-treated rats, as previously mentioned. However, SRL treatment after CsA therapy (Conversion protocol) was unable to restore basal levels of CTGF, suggesting that, once again, some of the lesions induced by CsA are maintained after the conversion for SRL.

In chronic kidney disease, rapamycin was able to slow the progression of renal fibrosis and delayed the onset of renal failure, through reduction of glomerular hypertrophy, decrease of proinflammatory and profibrotic cytokines production, and decline in interstitial inflammation [48]. As previously suggested, rapamycin is less fibrogenic than CsA [20], which is in agreement with the reduced kidney damage in the conversion protocol of our study. Our results reinforce the rationale for the early substitution of CsA by SRL, not only because longer CsA exposure is notoriously more deleterious, promoting structural kidney deterioration, but also because mTOR overexpression seems to be a feature of the chronic CsA exposure.

## 5. Conclusions

This experimental study demonstrated that CsA-induced nephrotoxicity is significantly aggravated over time and distinct mechanisms seem to underlie short- and long-term renal toxicity. The currently used clinical techniques and biomarkers, namely of biochemical impairment (such as serum and urine creatinine and BUN contents and clearance), if coupled with genetic and protein analysis in different samples, will bring more accuracy to early detect and follow up the appearance and development of nephrotoxicity. Conversion to SRL prevented CsA-induced renal damage evolution, which is better viewed by nontraditional, emergent biomarkers including serum TGF-*β* and IL-7, TBARs clearance, and kidney TGF-*β* and mTOR, while NGAL (serum versus urine) seems to be a feasible indicator of substitution to the mTOR inhibitor.
